# Intestinal *Cetobacterium* and acetate modify glucose homeostasis via parasympathetic activation in zebrafish

**DOI:** 10.1080/19490976.2021.1900996

**Published:** 2021-04-12

**Authors:** Anran Wang, Zhen Zhang, Qianwen Ding, Yalin Yang, Jérôme Bindelle, Chao Ran, Zhigang Zhou

**Affiliations:** a Department of AgroBioChem/Precision Livestock and Nutrition Unit, AgroBioChem/TERRA, Gembloux Agro-Bio Tech, Liège University (ULiège), Gembloux, Belgium; b China-Norway Joint Lab on Fish Gastrointestinal Microbiota, Feed Research Institute, Chinese Academy of Agricultural Sciences, Beijing China; c Key Laboratory for Feed Biotechnology of the Ministry of Agriculture, Feed Research Institute, Chinese Academy of Agricultural Sciences, Beijing China; d Norway-China Joint Lab on Fish Gastrointestinal Microbiota, Institute of Biology, Norwegian University of Science and Technology, Trondheim, Norway

**Keywords:** Gut microbiota, *Cetobacterium*, acetate, glucose homeostasis, zebrafish

## Abstract

The capability of carbohydrate utilization in fish is limited compared to mammals. It has scientific and practical significance to improve the ability of fish to use carbohydrates. The efficiency of dietary carbohydrate utilization varies among fish with different feeding habits, which are associated with differential intestinal microbiota. In this study, we found that zebrafish fed with omnivorous diet (OD) and herbivorous diet (HD) showed better glucose homeostasis compared with carnivorous diet (CD) fed counterpart and the differential glucose utilization efficiency was attributable to the intestinal microbiota. The commensal bacterium *Cetobacterium somerae*, an acetate producer, was enriched in OD and HD groups, and administration of *C. somerae* in both adult zebrafish and gnotobiotic larval zebrafish models resulted in improved glucose homeostasis and increased insulin expression, supporting a causative role of *C. somerae* enrichment in glucose homeostasis in fish. The enrichment of *C. somerae* was constantly associated with higher acetate levels, and dietary supplementation of acetate promotes glucose utilization in zebrafish, suggesting a contribution of acetate in the function of *C. somerae*. Furthermore, we found that the beneficial effect of both acetate and *C. somerae* on glucose homeostasis was mediated through parasympathetic activation. Overall, this work highlights the existence of a *C. somerae*-brain axis in the regulation of glucose homeostasis in fish and suggests a role of acetate in mediating the axis function. Our results suggest potential strategies for improvement of fish carbohydrate utilization.

## Introduction

Carbohydrates are among the important metabolic functional nutrients in fish, and it is also a kind of relatively cheap energy source in aquatic feed. Numerous studies have proven that the utilization of carbohydrates in fish is limited compared to mammals, especially in carnivorous species. It has been reported that fish fed with high levels of carbohydrates will lead to persistent postprandial hyperglycemia, and further induce low efficiency of feed utilization, abnormal fat deposition, and even high mortality.^1–3^ Therefore, effective improvement of the ability of fish to use carbohydrates is an urgent scientific problem in aquaculture. Although metformin has been reported to be an antidiabetic drug which can improve fish glucose homeostasis after dietary carbohydrate intake, metformin was also found to induce gluconeogenic and hepatic lipogenic gene expression.^3–6^ In this sense, it is urgent to find safe and effective substances to improve the utilization of carbohydrates in fish.

Currently, hypotheses have been proposed to explain this glucose intolerance in fish, including differences in body temperature, numbers of insulin receptors, hormonal regulation, ability of hepatic lipogenesis from glucose and imbalance between hepatic glycolysis and gluconeogenesis.^7–11^ In most teleost, the efficiency of dietary carbohydrate utilization was also shown to be associated with feeding habits. Generally, omnivorous fish (common carp, Indian major carps, and many catfish) and herbivorous fish (Nile tilapia) are relatively tolerant to and can effectively use dietary non-starch polysaccharide carbohydrates. It has been proven that feeding habits could influence the gut microbiota in fish.^12,13^ The gut bacterial diversity is generally lower in carnivores, and progressively increases in omnivores and herbivores.

In mammals, the gut microbiota plays an important role in dietary carbohydrate metabolism.^14–16^ While starch is the only digestible polysaccharide, the gut microbiota synthesizes a broad spectrum of hydrolases that help the fermentation of complex dietary carbohydrates to short-chain fatty acids (SCFAs) such as acetate, propionate, and butyrate,^17^ which play a pivotal role in microbiota-gut-brain crosstalk.^18^ SCFAs can directly reach the brain and affect brain feeding centers, altering neuropeptide release, and modifying feeding behavior and energy homeostasis.^19^ A high number of studies have emphasized the critical impact of gut microbiota on regulating glucose tolerance, systemic insulin sensitivity, and host energy homeostasis via intestinal microbial metabolites, including SCFAs, bile acids, indole, and succinate.^20–24^ However, research in this field has mostly focused on mammals, and very few studies have been conducted on fish. Thus, study on fish models may provide novel insight into the specific mechanisms involved in the microbiota-mediated regulation of glucose homeostasis in fish, and help improve carbohydrate utilization in aquaculture practices.

As an important vertebrate animal model, zebrafish has been used in the research of fish nutrition metabolism and gut microbiota interactions with hosts. At present, there have been studies on the core gut microbiota and its composition during different development stages. In addition, the successful establishment of germ-free (GF) zebrafish model provides an important method for studying the interactions between microbiota and hosts. In this study, we demonstrated that gut microbiota played an important role in the effect of feeding habit on zebrafish glucose metabolism, and the enrichment of intestinal indigenous bacteria *Cetobacterium somerae* contributed to the microbiota-mediated effect. Furthermore, we evaluated the effect of *C. somerae* on zebrafish glucose homeostasis. Our results suggested a beneficial gut *Cetobacterium*-acetate-brain axis for regulating zebrafish blood glucose.

## Materials and methods

### Zebrafish husbandry and experimental diets

All experimental and animal care procedures were approved by the Feed Research Institute of Chinese Academy of Agricultural Sciences Animal Care Committee, under the auspices of the China Council for Animal Care (Assurance No. 2015-AF-FRI-CAAS-003).

In experiment 1, to explore the effect of feeding habit on the gut microbial composition and glucose homeostasis, 2-month-old zebrafish (n = 4 tanks/group, 18 fish per tank) were fed with carnivorous diet (CD), omnivorous diet (OD), and herbivorous diet (HD) for 2 weeks (Supplemental Table 1). In experiment 2 involving *Aeromonas veronii* B565 and *C. somerae*, 2-month-old zebrafish were fed basal diet with or without nonabsorbable antibiotic mixture (Polymyxin B 2.5 g/kg diet and Neomycin 3.3 g/kg diet) for 1 week (Supplemental Table 2). Antibiotic-treated group were further assigned into two groups. *C. somerae* or *A. veronii* B565 were applied daily to the rearing water at a final concentration of 10^3^ CFUs/ml for 2 weeks. During the treatment, zebrafish were fed with basal diet. Experiment 3 was a follow-up feeding trial to further investigate the mechanism of sodium acetate (NaAc) in regulating glucose homeostasis. One-month-old zebrafish were fed with control diet and NaAc supplemented diet (Sigma Chemical, 1.5 g/kg diet) (Supplemental Table 3) for 4 weeks. The dosage of NaAc was selected according to our previous research.^25^ In an assay with xylose and mannose, 2-month-old zebrafish (n = 3 tanks/group, 18 fish per tank) were fed with control diet, control diet containing xylose (100 g/kg diet) (Solarbio, Chemical) or mannose (10 g/kg diet) (Sinopharm, Chemical) for 2 weeks (Supplemental Table 4). The doses of xylose and mannose were determined according to the report of Kim et al.^26^ and our preliminary study (Supplemental Methods and Results), respectively.

During the feeding period, the water temperature was 28–30 °C (pH 6.8–7.5), and a 12 h/12 h light/dark cycle was used. Zebrafish were randomly assigned into 3-L tanks in the recirculating system and fed with the experimental diets at a ratio of 4% of their average body weight twice daily (08:30–09:00 am and 16:30–17:00 pm).

### Intestinal contents microbiota composition analysis

After 2 weeks diet intervention, the intestinal contents of zebrafish were collected 4 h after the last feeding under aseptic conditions. The intestinal content samples from six fish/tank were pooled as a replicate. Intestinal bacterial DNA was extracted by TIANamp Bacteria DNA Kit (TianGen, Beijing, China). The full-length of 16S rRNA was amplified using the primers 27 F (AGAGTTTGATCCTGGCTCAG) and 1493 R (GGTTACCTTGTTACGACTT) by PCR and sequenced in PacBio Sequel System using SMRT Cell protocol. Sequences were denoised using DADA2 and assembled into amplicon sequence variants (ASVs). A representative sequence of each ASV was assigned to a taxonomic level in the Ribosomal Database Project (RDP) database using the RDP classifier. Principal component analysis and heat-map analysis were performed by using R 3.1.0.

The quantitation of total bacteria and specific phylotype of adult zebrafish intestinal contents and larval zebrafish was determined by *q*PCR.^27^ Primer sets for universal bacteria or specific bacterial groups targeting the 16S rRNA gene are listed in Supplemental Table 5. For the adult zebrafish, results were expressed as Log10 copy numbers of bacterial 16S rDNA per milligram of intestinal contents. For the larval zebrafish, results were expressed as Log10 copy numbers of bacterial 16S rDNA per larva.

### Short-chain fatty acids (SCFAs) analysis

Intestinal content samples were collected from zebrafish 4 h post the last feeding. The intestinal contents from six fish were pooled. Twenty milligrams of content sample was lyophilized and resuspended with 0.15 ml of MeOH, respectively. Each sample was mixed vigorously with sonication for three times with 10 min. After sonication, the samples were centrifuged at 12,000 rpm for 10 min, and the supernatants were used for GC-MS analysis. GC-MS was performed on a GCMS-QP2010 Ultra with an autosampler (SHIMADZU) and the Rtx-wax capillary column (60 m, 0.25 mm i.d., 0.25 μm film thickness; SHIMADZU). Oven temperature was programmed from 60 to 100 °C at 5 °C/min, with a 1 min hold; to 150 °C at 5 °C/min, with a 5 min hold; to 225 °C at 30 °C/min, with a 20 min hold. Injection of a 2 μl sample was performed at 230 °C. Helium, at a flow of 1.2 ml·min^−1^, was the carrier gas. Electronic impact was recorded at 70 eV. Peak identity and internal response factors were determined using a 1-mM calibration cocktail including acetate, propionate, butyrate, isobutyrate, isovalerate and pentanoate. The weight of each lyophilized gut content sample was recorded for calibration.^27^

### Plasma glucose and insulin measurement

After 4–6 h of feeding, the zebrafish were anesthetized with ice water. The blood samples of the zebrafish were collected as previously described.^28^ Level of plasma glucose was measured using Glucose Assay Kit (Nanjing Jiancheng Bioengineering Institute, China) according to the manufacturer’s instructions. The level of plasma insulin in zebrafish was determined by Zebrafish INS Elisa Kit (mlbio, Shanghai, China) according to the manufacturer’s instructions. The plasma level of insulin in adult zebrafish was expressed as insulin units per liter (mIU/L).

### GF zebrafish generation and treatment

GF zebrafish were derived from normal zebrafish and reared following established protocols.^29^ We formulated microparticulate basal diet for zebrafish larvae (Supplemental Table S6). The microparticulate diet was sterilized by irradiation with 20 kGy gamma rays in an atomic energy center. Zebrafish larvae hatched from their chorions at 3 days post fertilization (dpf). Each group had six bottles with 30 fish per bottle. The transfer of gut microbiota of zebrafish (experiment 1) to germ-free zebrafish was performed according to the method described by Rawls et al.^30^ with minor modifications.^31,32^ The gut microbiota was added to a gnotobiotic zebrafish medium (GZM) containing 4 dpf GF zebrafish at a final concentration of 10^6^ CFU/ml of GZM. At 6 dpf, whole fish were collected for analysis of glucose and the expression of *insulin* gene. In experiment 2, *A. veronii* B565 + *Plesiomonas shigelloides* (at a ratio of 1:1), and *A. veronii* B565 + *P. shigelloides* + *C. somerae* (at a ratio of 1:1:1) were added to GZM containing 4 dpf GF zebrafish at a concentration of 10^6^ CFUs/ml, respectively. At 5 dpf, the yolk was largely absorbed and the GF zebrafish started feeding. At 6 dpf, whole fish samples were collected and rinsed with sterile GZM for analysis of microbiota, glucose, acetate levels, and the expression of *insulin* gene.

### Larval glucose and acetate measurement

At 6 dpf, pools of 15 larval zebrafish were collected in 1.5 ml microcentrifuge tubes and frozen at −80 °C after complete removal of water. Levels of free glucose were measured using High Sensitivity Glucose Assay Kit (Sigma, USA). For analysis, samples were resuspended in glucose assay buffer and homogenized using a tissue homogenizer (IKK). Reactions were assembled in black 96 well flat-bottom plates with clear bottoms (Corning Costar). Standard curves were generated using glucose standard solution and included in each assay. To correct for the background, include a sample blank for each sample by omitting the glucose enzyme mix. Reactions were incubated for 30 min at 37 °C in the dark. Fluorescence (excitation 535 nm; emission 587 nm) was measured using a Synergy microplate reader. The concentration of glucose was expressed as glucose content per larval zebrafish (pmol/larval zebrafish). Larval acetate was measured using Acetate Colorimetric Assay Kit (Sigma, USA) and examined at 450 nm. The acetate concentration was expressed as acetate content per larval zebrafish (nmol/larval zebrafish).

### Quantitative PCR analysis

At 6 dpf, pools of 20 larval zebrafish were collected in 1.5 ml microcentrifuge tubes and frozen at −80 °C after complete removal of water. Total RNA of larval zebrafish was extracted using a TRIzon Reagent (CWBIO, Beijing, China). The integrity of the total RNA was verified by visualization on a 1.2% agarose gel. RNA was dissolved in 50 ul RNase-free water and stored at −80 °C until use. The cDNA was synthesized by the FastKing gDNA Dispelling RT SuperMix (TianGen, Beijing, China) according to the manufacturer’s instructions. Additional dissociation curve analysis was performed and a single melting curve was observed in all cases. The *q*PCR was performed using the SYBR Green SuperReal PreMix Plus (TianGen, Beijing, China) on a Light Cycler 480 system (Roche). The primer of *insulin* is listed in Supplemental Table 5.

### Growth performance measurements

After feeding treatment (experiment 3), weight gain, feed conversion efficiency (FCE), daily feeding rate, and survival rate of zebrafish were calculated according to previous report.^32^Weight gain (%) = 100 × (final body weight – initial body weight)/initial body weight; Feed conversion efficiency (FCE) = weight gain of fish/food intake; Daily feeding rate (%/d) = 100 × total feed consumed/days × (initial body weight + final body weight)/2; Survival rate (%) = (number of fish at the end of the experiment/number of fish at the start of the experiment) ×100.

### Intracerebroventricular (ICV) injection of sodium acetate and atropine

Zebrafish were handled with hypothermic anesthesia, and the operation of ICV injection was performed under stereoscopic anatomic mirror as described by Barbosa et al.^33^ To investigate the mechanism of NaAc regulation of zebrafish glucose metabolism, zebrafish in the experimental group were injected with 75 mg/kg NaAc, 0.1 mg/kg of atropine (Solarbio, Chemical), 75 mg/kg NaAc + 0.1 mg/kg atropine in sterile saline after 24 h fasting, respectively. The control group was injected with an equivalent saline. At 4 h and 6 h post injection, levels of blood glucose and insulin were measured.

After *C. somerae* and *A. veronii* B565 treatment (experiment 2), zebrafish were randomly sorted into two groups per treatment. The control group was injected with an equivalent saline, and the experimental group were injected with 0.1 mg/kg of atropine.

The ICV injection doses of NaAc were calculated according to the addition ratio in diets and the daily feeding rate (nearly 4%) based on the above feeding experiment. The dose of atropine was according to previous study.^34^ The injection accuracy was confirmed by Evans blue dye (0.5 μl) in the ventricle.

### Statistical analysis

The statistical data reported include results from at least three biological replicates. All results are expressed as the mean ± standard errors of the means (SEMs). All statistical analyses were performed in GraphPad Prism 8 (GraphPad Software Inc., San Diego, CA, USA). Comparisons between two groups were analyzed using the Student’s *t*-test, and comparisons between multiple groups were analyzed using one-way ANOVA followed by a Duncan’s test. The statistical significance was set at *P*< .05.

## Results

### Feeding habit affects gut microbiota and glucose homeostasis in zebrafish

**Figure 1.**
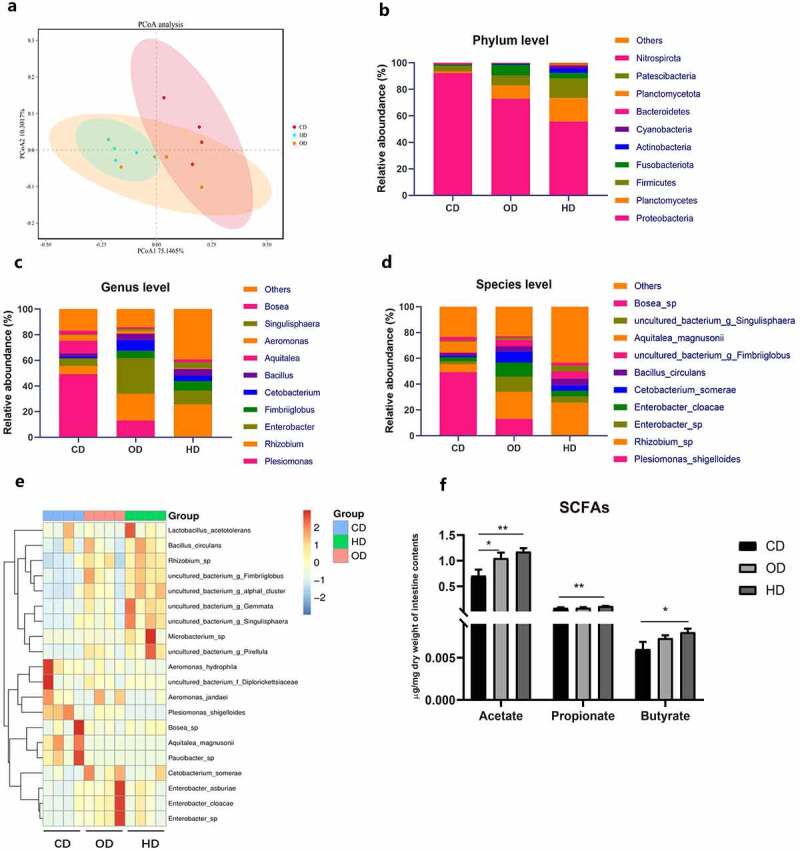
**Feeding habit affects gut microbiota and intestinal SCFAs in zebrafish**. Adult zebrafish (2-month-old) were fed with CD, OD and HD for 2 weeks. (a) Principal coordinate analysis (PCoA) of all samples by weighted UniFrac distance. The relative bacterial abundance at the phylum (b), genus (c) and species (d) levels of the gut microbiota of adult zebrafish. (e) Heatmap shows the relative abundance of relevant species. (f) Intestinal acetate levels, propionate levels and butyrate levels in zebrafish which was performed with GC-MS. Data were expressed as the mean ± SEM (n = 3 or 4 biological replicates). *p < .05; **p < .01

Firstly, we formulated carnivorous diet (CD), omnivorous diet (OD), and herbivorous diet (HD). After 2 weeks feeding, high-throughput sequencing of the full-length of 16S rRNA genes from intestinal contents was performed. Sequences were delineated into 1,067 amplicon sequence variants (ASVs). The Shannon and Simpson analysis showed differences in ASV diversity between CD and HD groups (Supplemental Table 7). Moreover, the overall structure of the gut microbiota in HD fed zebrafish significantly differed from that of CD group, as analyzed by principal coordinate analysis (PCoA) (Figure 1(a)).

Taxon-based analysis revealed marked changes in the gut microbial composition in response to diets representing different feeding habits. At the phylum level, the abundance of Planctomycetota, Firmicutes, Fusobacteria was significantly increased in OD and HD groups, and Proteobacteria was markedly decreased in OD and HD groups (Figure 1(b)). At the genus level, OD and HD enhanced the abundance of *Cetobacterium, Rhizobium, Enterobacter, Fimbriiglobus* and *Bacillus*, while *Plesiomonas* and *Aeromonas* were significantly reduced (Figure 1(c)). At the species level, *Cetobacterium somerae*, which belongs to Fusobacteria, was found to be enriched in OD and HD (relative abundance of 8.0% and 3.0%, respectively) treated groups compared with CD group (relative abundance of 1.0%), and *Plesiomonas shigelloides* was significantly decreased in HD group (Figure 1(d, e), Supplemental Table 8).

**Figure 2.**
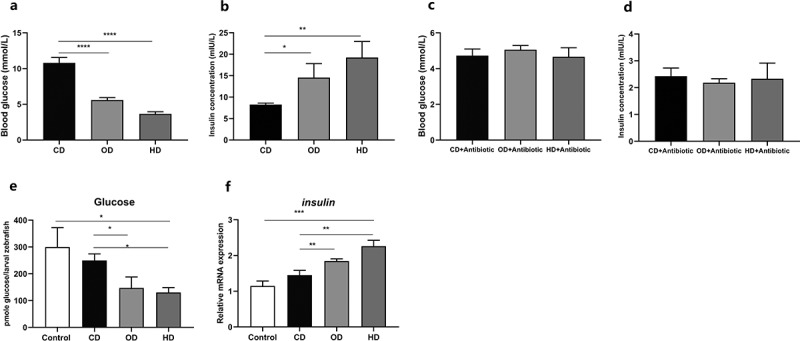
**Feeding habit affects glucose homeostasis in zebrafish**. Postprandial blood glucose (a) and insulin levels (b) in zebrafish. Adult zebrafish (2-month-old) were fed CD, OD and HD with antibiotic mixture (Polymyxin B 2.5 g/kg diet and Neomycin 3.3 g/kg diet) for 1 week. Postprandial blood glucose (c) and insulin (d) in zebrafish after antibiotic treatment. The intestinal microbiota of CD, OD and HD-treated zebrafish were transferred to GF zebrafish. Free glucose (e) and the relative expression of *insulin* (f) of GF zebrafish inoculated with intestinal bacteria of CD, OD and HD treated zebrafish. Data were expressed as the mean ± SEM (n = 3 biological replicates). *p < .05; **p < .01; ***p < .001; ****p < .0001

In line with the enrichment of acetate-producing *C. somerae*, we detected a significant increase in the acetate level in the intestinal contents of OD and HD treated group. HD treatment also significantly increased the level of propionate, butyrate, isobutyrate, pentanoate and isovalerate (Figure 1(f), Supplemental Figure 1(a-c)) compared with CD group. To investigate the influence of feeding habit on glucose homeostasis, we detected the postprandial blood glucose and insulin levels. We observed that OD and HD groups significantly decreased postprandial blood glucose levels and increased insulin concentration (Figure 2(a, b)).

To investigate the role of gut microbiota in the effect of feeding habit on glucose homeostasis in fish, we fed zebrafish with CD, OD and HD supplemented with nonabsorbable antibiotics (Polymyxin B 2.5 g/kg diet and Neomycin 3.3 g/kg diet) for 1 week. Antibiotic treatment severely reduced microbial abundance (Supplemental Figure 2(a-c)), and the effect of feeding habit on postprandial blood glucose and insulin was abrogated by antibiotic treatment (Figure 2(c, d)). To confirm the relationship between feeding habit-associated microbiota and glucose homeostasis, we transferred the intestinal microbiota of CD, OD and HD-fed zebrafish to GF zebrafish, and tested the whole larvae glucose level (Figure 2(e)) and the relative expression of *insulin* (Figure 2(f)). The results showed that the glucose and insulin phenotypes in GF zebrafish colonized with microbiota from different groups were consistent with the donor zebrafish. Taken together, these results suggest the intestinal microbiota plays a causative role in feeding habit associated difference in blood glucose homeostasis in zebrafish.

### Effect of C. somerae on zebrafish glucose homeostasis

**Figure 3.**
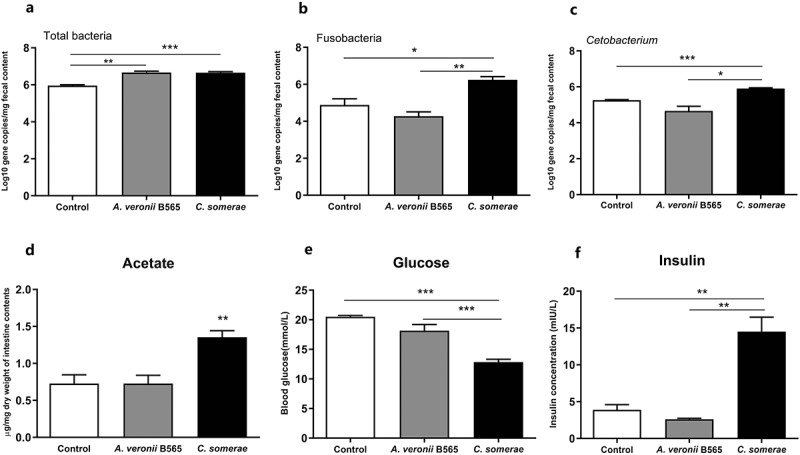
**Effect of *C. somerae* on glucose homeostasis in adult zebrafish**. Adult zebrafish (2-month-old) were fed basal diet with antibiotic mixture or without for 1 week. Then, the zebrafish fed with antibiotics were treated with *C. somerae* or *A. veronii* B565 for 2 weeks. Total number of bacteria (a), the number of Fusobacteria (b) and *Cetobacterium* (c) in the intestinal microbiota of zebrafish. Intestinal acetate (d), postprandial blood glucose (e) and insulin (f) in zebrafish. Data were expressed as the mean ± SEM (n = 3 biological replicates). *p < .05; **p < .01; ***p < .001

*C. somerae* showed large enrichment in OD and HD groups versus CD group. Our previous results suggest the beneficial effect of *C. somerae* in metabolic health of zebrafish.^26^ Therefore, we next investigated the potential contribution of *C. somerae* on glucose homeostasis. To confirm the beneficial and causative effects of the enriched *C. somerae*, conventionally raised (CONR) zebrafish were fed basal diet with or without nonabsorbable antibiotic mixture for 1 week. Then, *C. somerae* was applied to the rearing water of antibiotic-treated zebrafish to facilitate its enrichment. *A. veronii* B565, a commensal *Aeromonas* strain, was applied similarly to a negative control. We detected that *C. somerae* treatment increased the total bacteria quantity of intestinal contents (Figure 3(a)). Fusobacteria/*Cetobacterium* were significantly increased in *C. somerae*-treated zebrafish compared to the control and *A. veronii* B565 treatment (Figure 3(b, c)), while *Plesiomonas* and *Aeromonas* were decreased (Supplemental Figure 3(b, c)). Consistent with the acetate production activity of *C. somerae*, we found significantly increased SCFAs levels in zebrafish treated with *C. somerae* (Supplemental Figure 4(a-e)), especially acetate (Figure 3(d)). Furthermore, we also observed significantly decreased postprandial blood glucose levels (Figure 3(e)) and increased insulin concentration (Figure 3(f)) of *C. somerae* treated zebrafish.

**Figure 4.**
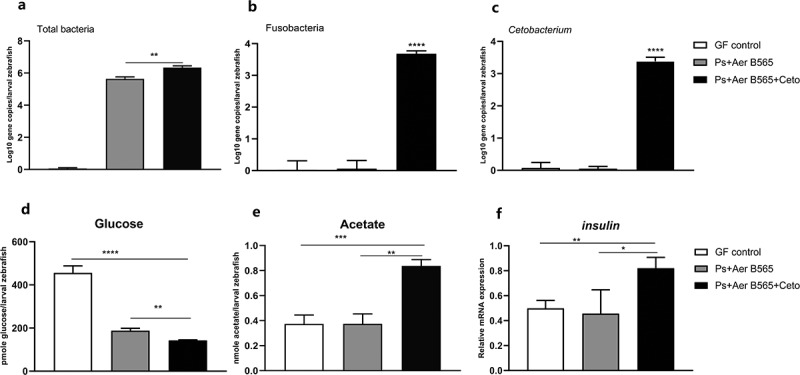
**Effect of *C. somerae* on glucose homeostasis in larval zebrafish**. 4 dpf GF zebrafish were inoculated with *A. veronii* B565 *+ P. shigelloides*, and *A. veronii* B565 *+ P. shigelloides* *+* *C. somerae* for 2 weeks. Total number of bacteria (a), the number of Fusobacteria (b) and *Cetobacterium* (c) of the microbiota of zebrafish larvae at 6 dpf. Free glucose (d), acetate (e) and the relative expression of *insulin* (f) of zebrafish larvae at 6 dpf. Data were expressed as the mean ± SEM (n = 3 biological replicates). *p < .05; **p < .01; ***p < .001; ****p < .0001

To further confirm the effect of *C. somerae* on glucose homeostasis, we innoculate GF zebrafish with *A. veronii* B565 *+* *P. shigelloides*, and *A. veronii* B565 *+* *P. shigelloides* *+* *C. somerae* at 4 dpf. Similarly, the colonization of *A. veronii* B565, *P. shigelloides* and *C. somerae* was confirmed by *q*PCR determination (Figure 4(a-c), Supplemental Figure 3(d-f)). Furthermore, we measured the glucose levels of 6 dpf whole-larvae using an enzymatic assay that detects free glucose. Consistently, gnotobiotic zebrafish with *C. somerae* colonization showed significantly lower glucose levels than GF control zebrafish and zebrafish colonized with representative Proteobacteria species (Figure 4(d)), and *C. somerae* colonization was associated with higher acetate level in whole-larvae zebrafish (Figure 4(e)). Moreover, the expression of *insulin* gene was up-regulated in zebrafish colonized with *C. somerae* (Figure 4(f)). Altogether, these findings demonstrate the regulation of zebrafish glucose homeostasis by commensal *C. somerae* and suggest a role for its metabolite acetate in this regulation.

### Sodium acetate treatment have effect on zebrafish glucose homeostasis

**Figure 5.**
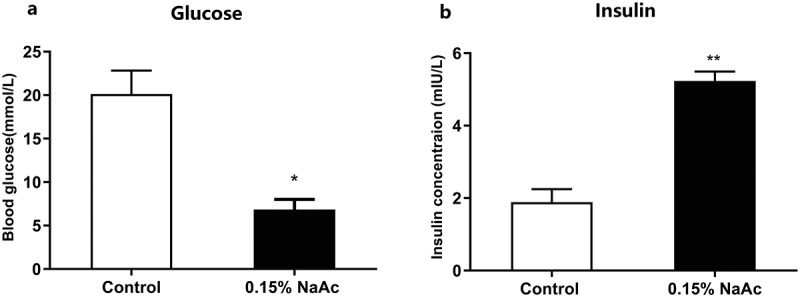
**Dietary sodium acetate promotes glucose homeostasis in zebrafish**. Postprandial blood glucose (a) and insulin (b) of zebrafish fed with control and 0.15% NaAc diets for 4 weeks. Data were expressed as the mean ± SEM (n = 3 biological replicates). *p < .05; **p < .01

In order to further evaluate the effects of acetate on glucose metabolism, zebrafish were fed with 0.15% NaAc diet at a ratio of 4% of body weight for 4 weeks. The treatment caused significantly enhanced body weight gain and FCE compared to the control group (Supplemental Figure 5(a, b)), and the daily feeding rate was significantly lower than the control group (Supplemental Figure 5(c)). These results indicated that increased weight gain induced by NaAc has no correlation to food intake. Next, we evaluate whether NaAc diet has an effect on the postprandial blood glucose and insulin secretion. We observed a significantly decreased blood glucose level and increased insulin concentration in NaAc fed zebrafish (Figure 5(a, b)).

### Sodium acetate drives insulin secretion via the parasympathetics

**Figure 6.**
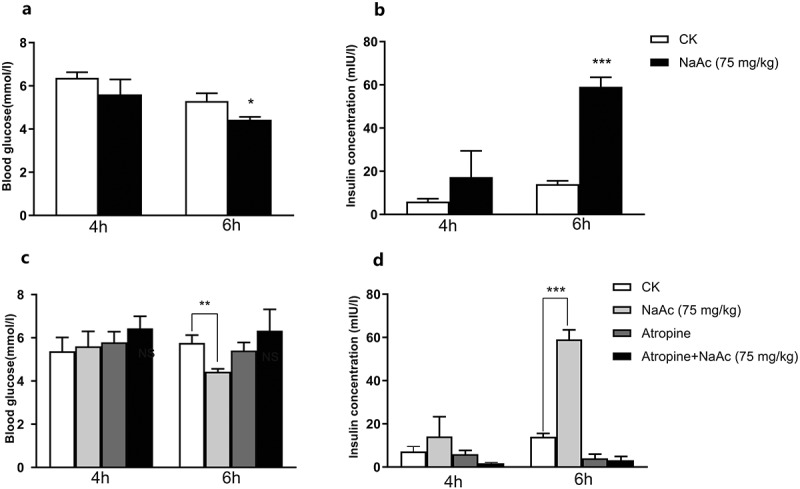
**Sodium acetate drives insulin secretion via parasympathetic activation**. Postprandial blood glucose (a) and insulin (b) of zebrafish after ICV injection of saline or 75 mg/kg NaAc at 4 h and 6 h. Postprandial blood glucose (c) and insulin (d) of zebrafish after ICV injection of saline, 75 mg/kg NaAc, 0.1 mg/kg atropine, or 75 mg/kg NaAc + 0.1 mg/kg atropine at 4 h and 6 h. Data were expressed as the mean ± SEM (n = 3 biological replicates). *p < .05; **p < .01; ***p < .001

Having found a causal relationship between acetate and insulin secretion, we next examined the underlying mechanism. It has been reported that acetate increased glucose-stimulated insulin secretion (GSIS) in rats by activation of the parasympathetic nervous system.^35^ To investigate whether acetate functions via parasympathetic activation in zebrafish, we performed intracerebroventricular (ICV) injection of NaAc at a dose of 75 mg/kg. ICV injection of NaAc in zebrafish tripled the increase in insulin measured at 6 h after injection and decreased the blood glucose levels (Figure 6(a, b)), while this was blocked when NaAc was co-injected with parasympathetic blocker atropine (Figure 6(c, d)). These results indicated that the effect of acetate on glucose homeostasis in zebrafish was mediated by parasympathetic activation. Furthermore, the effects of *C. somerae* were also blocked after ICV injection of atropine in *C. somerae* treated zebrafish (Supplemental Figure 6(a, b)). This indicated that the function of *C. somerae* was also mediated by parasympathetics, supporting a *C. somerae*-brain axis in the regulation of glucose homeostasis in fish. Moreover, these results suggest that the effect of commensal *C. somerae* on glucose homeostasis was mediated by acetate production.

### Mannose promotes the enrichment of gut Cetobacterium in zebrafish

**Figure 7.**
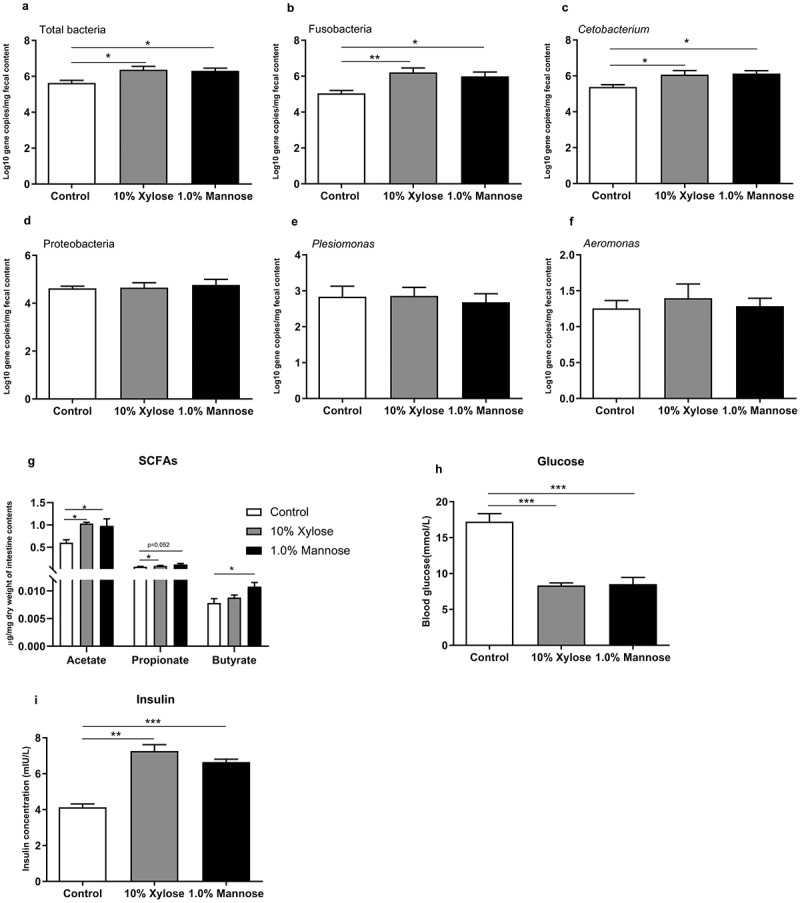
**Mannose promotes the enrichment of gut *Cetobacterium* in zebrafish**. Adult zebrafish (2-month-old) were fed with control diet, 10% xylose diet or 1.0% mannose diet for 2 weeks. Total number of bacteria (a), the number of Fusobacteria (b), *Cetobacterium* (c), Proteobacteria (d), *Plesiomonas* (e) and *Aeromonas* (f) in the intestinal microbiota of zebrafish. Intestinal acetate (g), postprandial blood glucose (h) and insulin (i) in zebrafish. Data were expressed as the mean ± SEM (n = 3 or 4 biological replicates). *p < .05; **p < .01; ***p < .001

Since *C. somerae* is anaerobic bacteria, there are some limitations in practical application. Therefore, we try to explore whether prebiotics could be used to promote *Cetobacterium* enrichment in zebrafish. *In vitro* tests have demonstrated that xylose and mannose could significantly promote *Cetobacterium* growth and depress the growth of *Plesiomonas* (Supplemental Table 9). We next verified whether xylose and mannose could enrich *Cetobacterium in vivo*. We compared the gut microbiota composition of zebrafish fed with 10% xylose or 1.0% mannose. After 2 weeks feeding trial, we found that xylose and mannose supplementation significantly increased the abundance of total bacteria, Fusobacteria and *Cetobacterium* compared to the control (Figure 7(a-c)), while both xylose and mannose had no effect on the abundance of Proteobacteria, *Plesiomonas* and *Aeromonas* (Figure 7(d-f)), suggesting that xylose and mannose favored the growth of Fusobacteria and *Cetobacterium in vivo*.

Next, we tested the SCFAs levels in zebrafish intestine. Both xylose and mannose significantly increased the levels of acetate and propionate compared to the control group (Figure 7(g)). However, butyrate, isobutyrate, pentanoate and isovalerate are only increased by mannose (Figure 7(g), Supplemental Figure 7(a-c)). Furthermore, we also observed significantly decreased postprandial blood glucose level and increased insulin concentration in zebrafish fed xylose and mannose supplemented diets (Figure 7(h, i)).

## Discussion

Glucose metabolism and homeostasis are highly dependent on feeding status,^7^ and the ability of carbohydrate utilization exhibited discrepancy in fish of different feeding habit. Many carnivorous fish exhibit persistent postprandial hyperglycemia after feeding digestible carbohydrates.^1,3,36^ In contrast, the omnivorous common carp (*Cyprinus carpio*) and Nile tilapia (*Oreochromis niloticus*) were able to use high levels of carbohydrates (50% in the diet).^1,36,37^

The feeding habit of fish influences the structure of intestinal microbiota. In this study, we use omnivorous zebrafish as a fish model and investigate the role of gut microbiota in the effect of feeding habit on glucose homeostasis in fish. Zebrafish were fed three formulated diets mimicking carnivorous diet (CD), omnivorous diet (OD), and herbivorous diet (HD). It has been reported that the composition of intestinal microbiota was influenced by feeding habit.^12^ In this study, zebrafish fed CD, OD, and HD exhibited different gut microbiota structure. Notably, the abundance of *C. somerae* increased in zebrafish fed OD and HD, while the abundance of *P. shigelloides* decreased (Figure 1(d), Supplemental Table 8). Generally, the intestinal microbiota of fish is largely different from that of in mammals, with Fusobacteria and Proteobacteria dominant in fish gut rather than Firmicutes and Bacteroidetes. In particular, *C. somerae* is the main species constituting the phylum of Fusobacteria in fish microbiota. *C. somerae* has been identified in the microbiota of many fish species.^38–42^ Among zebrafish fed CD, OD and HD, the abundance of *C. somerae* showed a positive correlation with glucose homeostasis in fish, suggesting a beneficial effect of enriched *C. somerae* on glucose utilization. Consistently, administration of *C. somerae* in both adult zebrafish and gnotobiotic larval zebrafish models resulted in improved glucose homeostasis and increased *insulin* expression (Figure 3(e, f), Figure 4(d, f)), supporting a causative correlation between *C. somerae* abundance and glucose homeostasis in fish. Besides *C. somerae*, the abundance of *Rhizobium, Enterobacter, Fimbriiglobus*, and *Bacillus* was also increased in HD and OD groups compared to CD groups. While our results confirmed the beneficial effect of *C. somerae* on glucose homeostasis in fish, we cannot rule out potential benefits mediated by other bacterial species, which deserves further investigation.

Consistent with previous results,^43^ we found that *C. somerae* produces a high amount of acetate, and only a minor of propionate and butyrate (Supplemental Figure 8). Furthermore, exogenous *C. somerae* could stably colonize in zebrafish intestine *in vivo* (Figure 3(c)), and increase the intestinal level of acetate, propionate, and butyrate (Figure 3(d), Supplemental Figure 4(a, b)). Studies have indicated that acetate can promote glucose-stimulated insulin secretion,^35,44^ while propionate and butyrate have no such effect.^45^ Consistent with the results in mammals, we confirmed that dietary acetate supplementation promoted insulin secretion and glucose utilization in zebrafish (Figure 5(a, b)), indicating a conserved role of acetate in regulating glucose homeostasis. The abundance of *C. somerae* and intestinal acetate levels are highly correlated in both adult and gnotobiotic zebrafish (Figure 3(d), Figure 4(e)), suggesting that the positive effect of *C. somerae* on glucose homeostasis in zebrafish is mediated by acetate production. Notably, our results cannot rule out the potential contribution of other SCFAs, such as isobutyrate, to the benefits of *C. somerae*, but support that acetate was at least partially responsible for the beneficial effect.

The mechanisms underlying the role of acetate in the regulation of insulin secretion and glucose utilization have been investigated in mammals. Perry et al.^35^ reported that acetate promoted glucose-stimulated insulin secretion by activation of the parasympathetic nervous system. Interestingly, our results showed that the effect of acetate in zebrafish was also mediated by action on the parasympathetic nervous system (Figure 6(a, b)), indicating that the acetate-brain-insulin secretion axis is conserved between fish and mammals. Furthermore, we found that the effect of *C. somerae* on insulin secretion and glucose utilization can be abrogated by blocking the parasympathetic nervous system (Figure 6(c, d)), supporting that the effect of *C. somerae* was mediated by acetate production.

In humans and mammals, insulin is the only pancreatic cell hormone known to directly regulate blood glucose.^46–49^ Mullapudi et al.^50^ has confirmed that insulin in zebrafish is also as important as in humans and mammals. Prior studies have demonstrated that the development of zebrafish cell is highly conserved with that of mammals. Hill et al.^51^ have shown that a specific protein produced by commensal *Aeromonas* sp., named BefA, promotes pancreas cell proliferation during larval zebrafish development. However, the role of other commensal bacteria and the bacterial metabolites in glucose homeostasis has never been investigated. Here, we use both adult and gnotobiotic zebrafish models to demonstrate the crucial role for *C. somerae* in controlling glucose homeostasis in zebrafish. Higher blood insulin level in adult zebrafish or *insulin* expression in larvae was observed in *C. somerae*-treated zebrafish.

In summary, we demonstrated that feeding habits have an influence on the glucose metabolism. OD and HD significantly decreased the postprandial blood glucose, and increased insulin secretion. An altered gut microbiota with increased abundance of acetate-producing *Cetobacterium* played a causative role in the improved glucose homeostasis in fish. Our discovery reveals a specific gut *Cetobacterium*-brain pathway for regulating zebrafish glucose homeostasis and suggests a role of acetate in mediating the function. Our results may provide insight into strategies for improvement of fish carbohydrate utilization.

**Table:** 

**Abbreviations**	
ASVs	amplicon sequence variants
CD	carnivorous diet
CONR	conventionally raised
FCE	feed conversion efficiency
GF	germ-free
GSIS	glucose-stimulated insulin secretion
GZM	gnotobiotic zebrafish medium
HD	herbivorous diet
ICV	intracerebroventricular
OD	omnivorous diet
PCoA	principal coordinate analysis
RDP	ribosomal database project
SCFAs	short-chain fatty acids

## Supplementary Material

Supplemental Material

## Data Availability

The 16S rRNA sequence raw data from this study were deposited in the National Center for Biotechnology Information (NCBI) Sequence Read Archive (SRA) under BioProject number PRJNA695108.
