# Supplementary material for: Intestinal Cetobacterium and acetate modify glucose homeostasis via parasympathetic activation in zebrafish
Source: Gut Microbes. 2021 Apr 12;13(1):1900996. doi: 10.1080/19490976.2021.1900996 (PMC8043178; doi:10.1080/19490976.2021.1900996)
Supplement: Supplemental Material [file KGMI_A_1900996_SM6815.docx]

**Supplemental Methods**

***Biochemical reaction analysis of C. somerae***

Four indigenous gut bacteria, *C. somerae, P. shigelloides, Aeromonas hydrophila* NJ-1, *Aeromonas hyrophila* CI098, were cultured on Glfu Anaerobic Medium (GAM) solid medium for 12 h at 28 ˚C. Then selected the single colony and put into the bacterial biochemical micro-identification tube (Hangzhou Binhe microbial Reagent Co., Ltd.) to identify its utilization of 26 kinds of carbohydrate substrates. Each substrate was set with 2 parallel plates and cultured at 28 ˚C for 24-48 h.

***Determination of short-chain fatty acids (SCFAs)-derived from C. somerae***

*C. somerae* was cultured in GAM medium for 12 h and 24 h at 28 ˚C under anaerobic and microaerophilic (7% oxygen) conditions. 0.5 ml of broth culture of *C. somerae* was lyophilized and resuspended with 1 ml of MeOH. Each sample was mixed vigorously with sonication for three times with 10 min. After sonication, the samples were centrifuged at 12,000 rpm for 10 min, and the supernatants were used for GC-MS analysis as described in Materials and methods.

***Dose effect of mannose on zebrafish glucose homeostasis***

To evaluate the dose effect of mannose on glucose homeostasis, 2-month-old zebrafish (n = 3 tanks/group, 18 fish per tank) were fed with control diet, control diet supplemented with mannose at different concentrations (0.25%, 0.5% and 1.0%) for 2 weeks. At the end of feeding trial, weight gain, feed conversion efficiency (FCE) and daily feeing rate of zebrafish were calculated according to previous report. ^32^ Moreover, postprandial blood glucose and insulin were also measured as described in Materials and methods.

**Supplemental Results**

***In vitro evaluation of C. somerae***

In order to gain more information about the biochemical character of *C. somerae,* we next performed *in vitro* tests to investigate the growth and ability of *C. somerae* to produce SCFAs. Biochemical reaction experiments were carried out to identify the utilization of different carbohydrates (alcohols and glycosides) by *C. somerae*, *P. shigelloide**s*, [*A.*](javascript:;)*hydrophila*NJ-1 and *A. veronii* CI098*.* As shown in Supplemental Table 9, xylose and mannose could promote the growth of *C. somerae*, and depressed *P. shigelloides* growth. Furthermore, we performed metabolic profiling experiments to determine the set of SCFAs produced by *C. somerae*. It has been detected that *C. somerae* produces highly abundant acetate up to 1500 μg/ml at 24 h under anaerobic condition, followed by propionate, isobutyrate, butyrate, isovalerate and pentanoate (Supplemental Figure 8(a-f)). Moreover, compared with microaerophilic group, the levels of acetate, propionate and isobutyrate produced by *C. somerae* were significantly higher under anaerobic condition.

***Effects of dietary mannose on zebrafish growth performance and glucose homeostasis***

After 2 weeks diet intervention, weight gain was significantly lower in zebrafish fed 0.5% and 1.0% mannose diets compared with control (Supplemental Figure 9(a)), while FCE and daily feeding rate were significantly enhanced (Supplemental Figure 9(b, c)). Furthermore, zebrafish fed with 1.0% mannose showed significantly decreased postprandial blood glucose and increased insulin concentration compared to control group (Supplemental Figure 9(d, e)).

**Supplemental Figures**


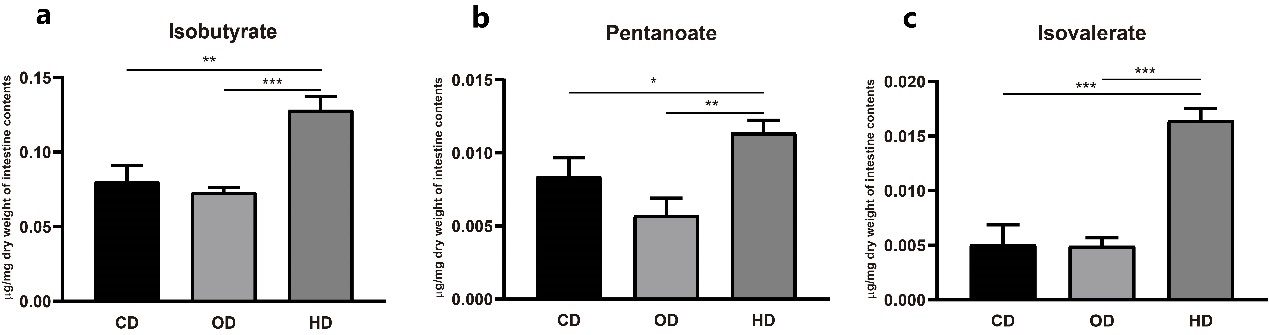


**Supplemental Figure 1. Effects of feeding habit on intestinal SCFAs in zebrafish.** Intestinal isobutyrate levels (a), isovalerate levels (b) and pentanoate levels (c) of zebrafish fed with CD, OD and HD for 2 weeks (n = 3 biological replicates). Data were expressed as the mean ± SEM. **p* < 0.05; ***p* < 0.01; ****p* < 0.001.


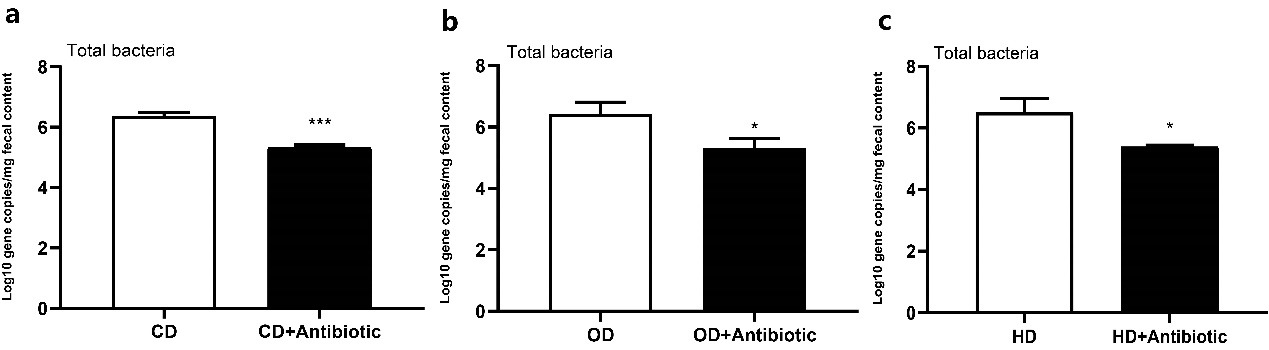


**Supplemental Figure 2. The efficiency of antibiotic mixture supplemented in CD, OD and HD.** (a-c) Total number of bacteria in the intestinal microbiota of zebrafish fed CD, OD and HD with antibiotic mixture or without for 1 week. Data were expressed as the mean ± SEM (n = 3 biological replicates). **p* < 0.05; ***p* < 0.01; ****p* < 0.001.


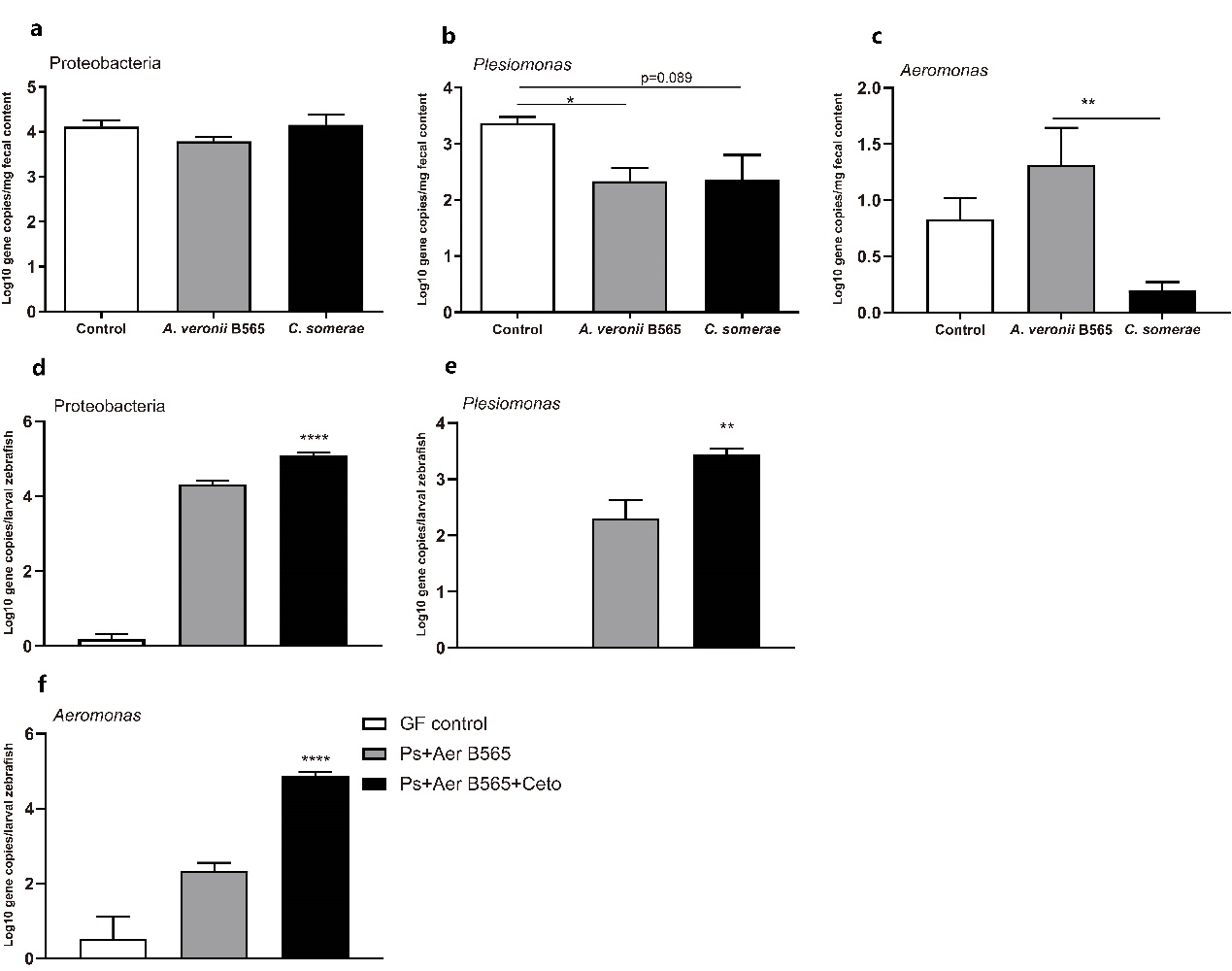


**Supplemental Figure 3. Effects of *C. somerae* on bacteria from Proteobacteria in adult zebrafish intestine and larval zebrafish.** The number of Proteobacteria (a), *Plesiomonas* (b) and *Aeromonas* (c) in the intestinal microbiota of zebrafish treated with *C. somerae* and *A. veronii* B565 for 2 weeks. The number of Proteobacteria (d), *Plesiomonas* (e) and *Aeromonas* (f) of the microbiota of zebrafish larvae inoculated with *A. veronii* B565, *P. shigelloides*, *C. somerae* at 6 dpf. Data were expressed as the mean ± SEM (n = 3 biological replicates). **p* < 0.05; ***p* < 0.01; ****p* < 0.001; *****p* < 0.0001.


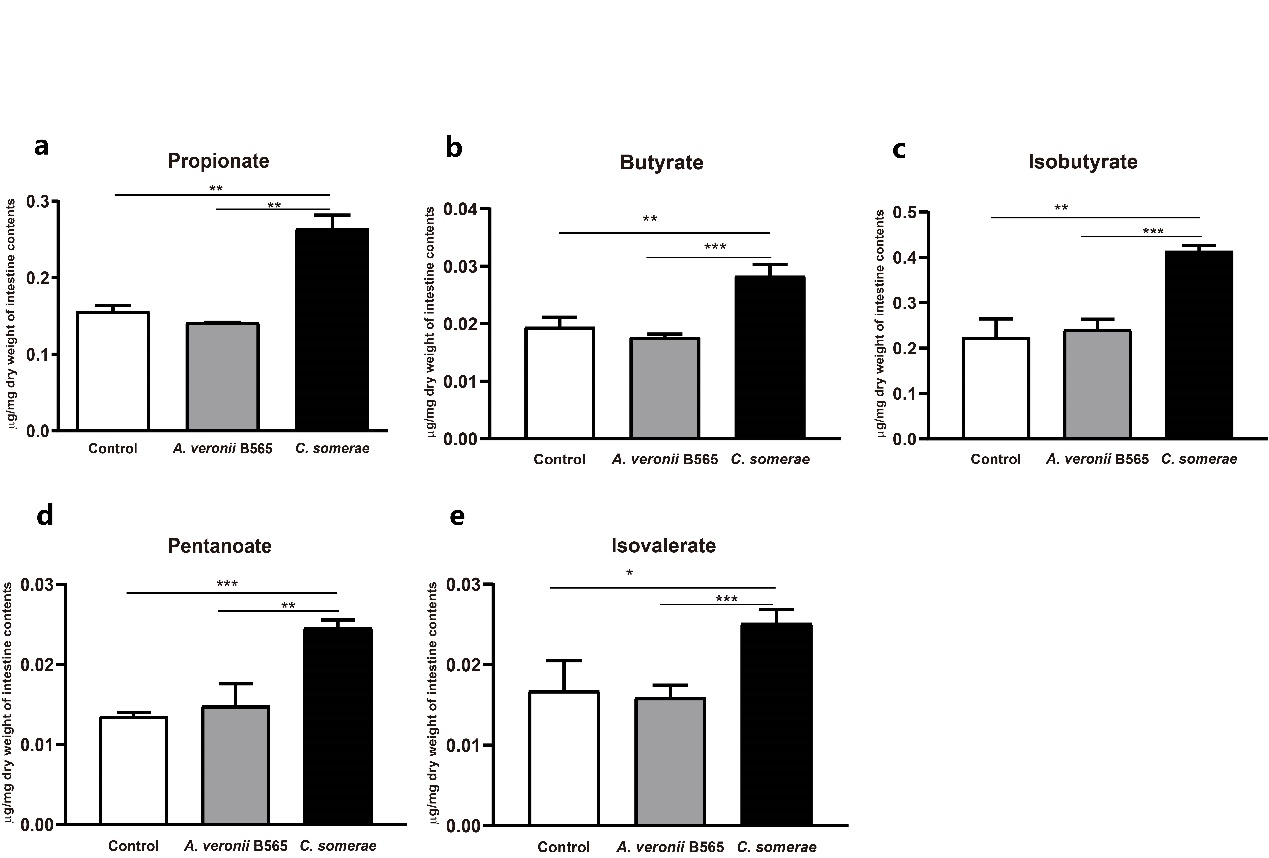


**Supplemental Figure 4. Effects of *C. somerae* on intestinal SCFAs in zebrafish.** Intestinal propionate levels (a), butyrate levels (b), isobutyrate levels (c), pentanoate levels (d) and isovalerate levels (e) in zebrafish treated with *C. somerae* and *A. veronii* B565 for 2 weeks. Data were expressed as the mean ± SEM (n = 3 biological replicates). **p* < 0.05; ***p* < 0.01; ****p* < 0.001.


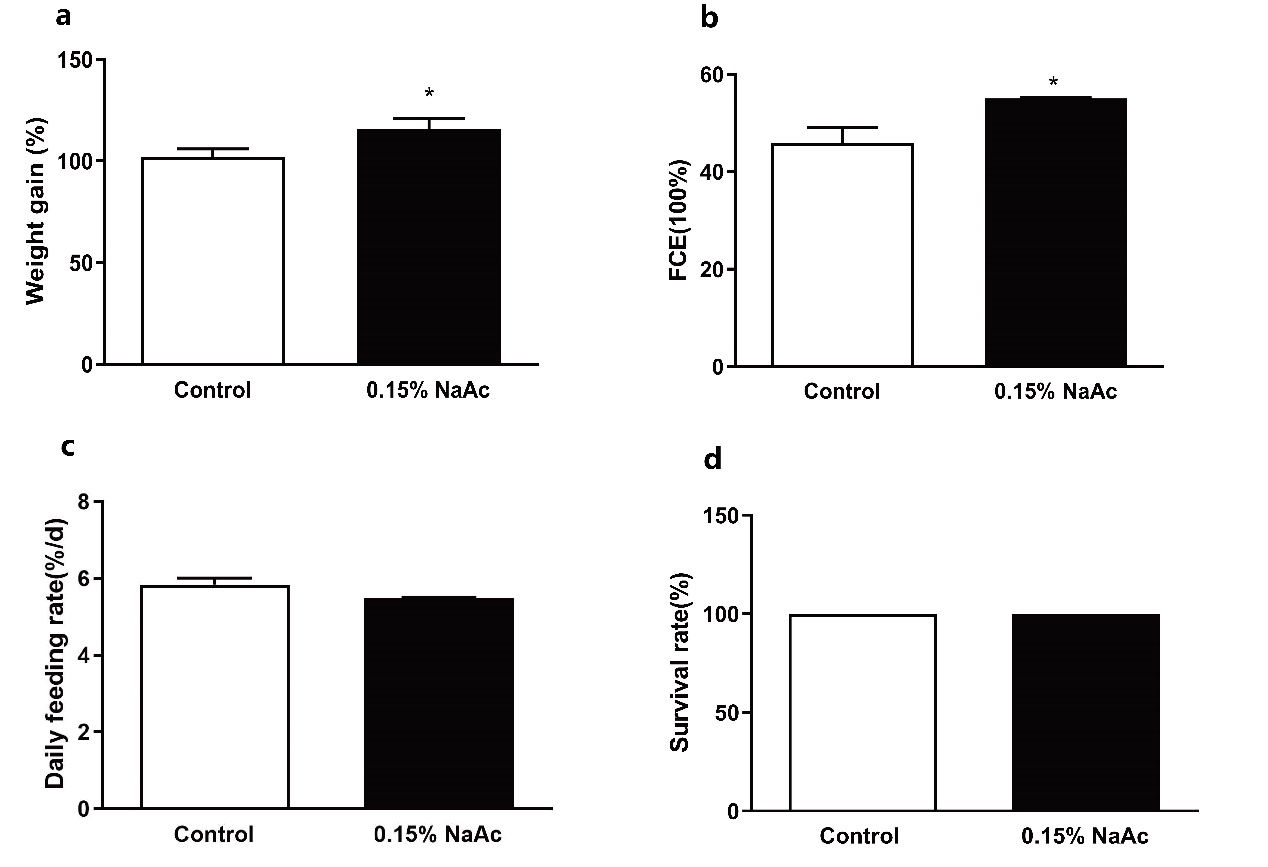


**Supplemental Figure 5. Effects of sodium acetate administration on the growth performance in zebrafish.** Adult zebrafish (one-month-old) were fed with the control and 0.15% NaAc diets for 4 weeks. The body weight gain (a), FCE (b), daily feeding rate (c) and survival rate (d) of adult zebrafish. Data were expressed as the mean ± SEM (n = 3 biological replicates). **p* < 0.05.


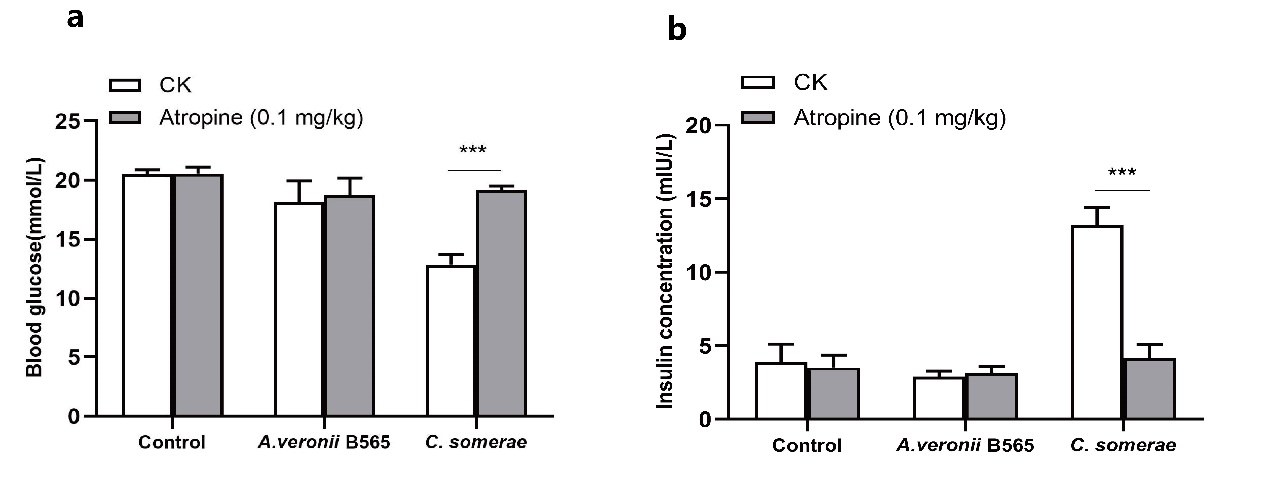


**Supplemental Figure 6. Effect of *C. somerae* on zebrafish glucose homeostasis was mediated by acetate.** In experiment 2, the *C. somerae* and *A. veronii* B565 treated zebrafish were randomly sorted into 2 groups per treatment, and ICV injected with an equivalent saline and 0.1 mg/kg atropine. Postprandial blood glucose (a) and insulin (b) in zebrafish. Data were expressed as the mean ± SEM (n = 3 biological replicates). ****p* < 0.001.


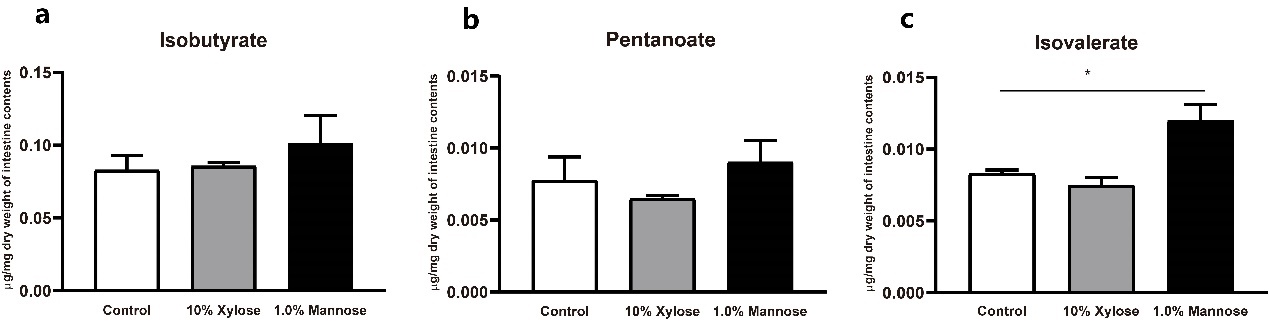


**Supplemental Figure 7. Effects of xylose and mannose on the intestinal SCFAs in zebrafish.** Intestinal isobutyrate levels (a), pentanoate levels (b) and isovalerate levels (c) of zebrafish fed with control diet, 10% xylose or 1.0% mannose diets for 2 weeks. Data were expressed as the mean ± SEM (n = 3 biological replicates). **p* < 0.05.


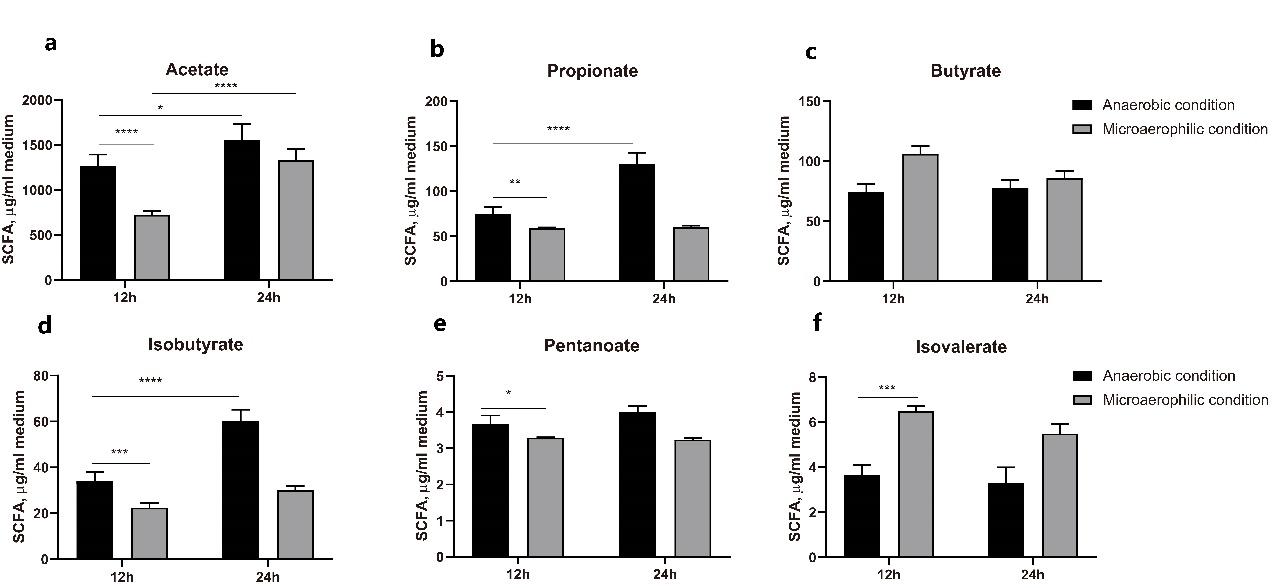


**Supplemental Figure 8. SCFAs produced by *C. somerae.*** Acetate levels (a), propionate levels (b), butyrate levels (c), isobutyrate levels (d), pentanoate levels (e) and isovalerate levels (f) produced by *C. somerae* which were cultured in GAM medium, and incubated at 28 ˚C for 12 h and 24 h under anaerobic and microaerophilic (7% oxygen) conditions. Data were expressed as the mean ± SEM (n = 3 biological replicates). **p* < 0.05; ***p* < 0.01; ****p* < 0.001.


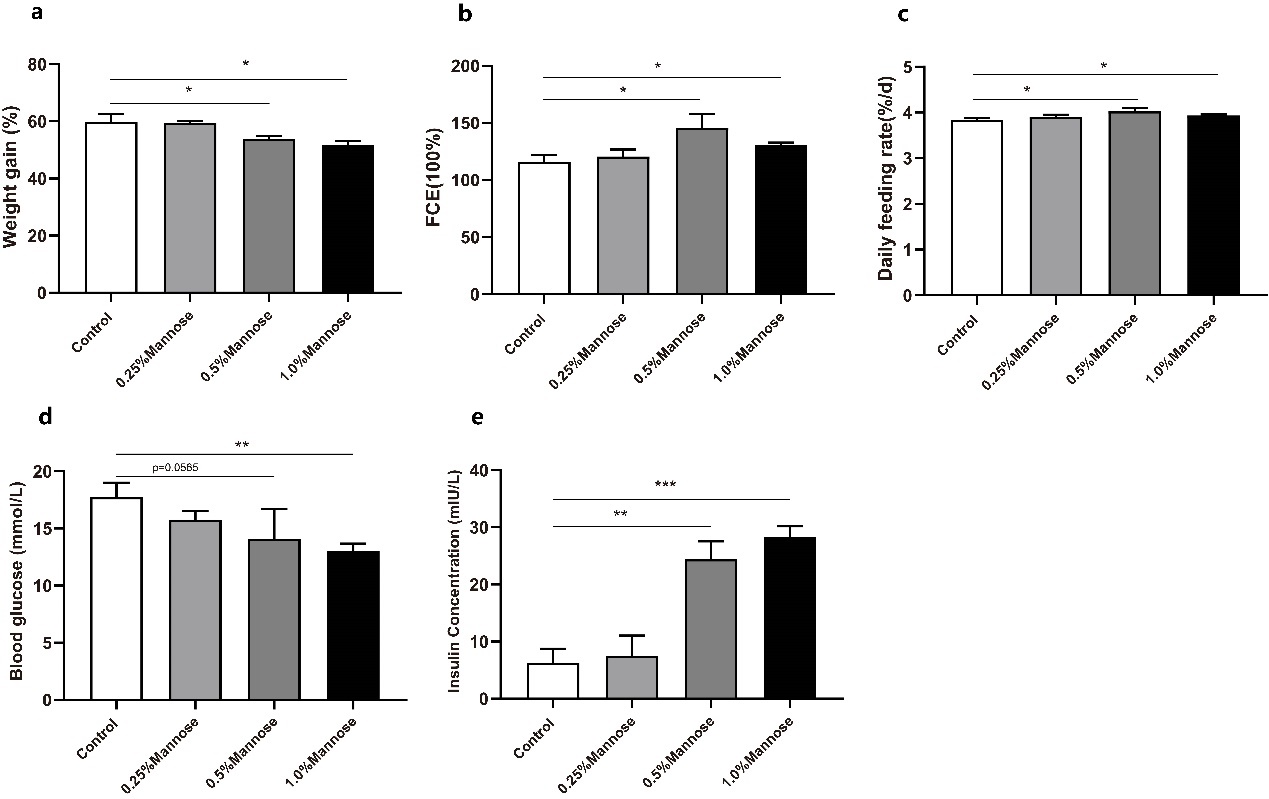


**Supplemental Figure 9. Dose effects of mannose.** Adult zebrafish (2-month-old) were fed on diets supplemented with mannose at different concentrations (0.00%, 0.25%, 0.5%, 1.0%) for 2 weeks. The body weight gain (a), FCE (b) and daily feeding rate (c) of adult zebrafish. Postprandial blood glucose (d) and insulin (e) in zebrafish. Data were expressed as the mean ± SEM (n = 3 biological replicates). **p* < 0.05; ***p* < 0.01; ****p* < 0.001.

**Supplemental Table 1. Ingredients and chemical compositions of diets for adult zebrafish (dry matter, g/kg diet).**

| Ingredient (g/kg diet) | Carnivorous diet | Omnivorous diet | Herbivorous diet |
| --- | --- | --- | --- |
| Soybean protein concentrate | 0.00 | 239 | 478 |
| Vital gluten | 0.00 | 50 | 100 |
| Casein | 400 | 200 | 0.00 |
| Gelatin | 10.00 | 50 | 0.00 |
| Dextrin | 280 | 277 | 273 |
| Soybean oil | 60 | 59 | 58 |
| Lysine | 3.3 | 10 | 16.7 |
| Methionine | 0.00 | 3.0 | 6.1 |
| VC phosphate | 1.0 | 1.0 | 1.0 |
| Vitamin premix^1^ | 4.0 | 4.0 | 4.0 |
| Mineral premix^2^ | 4.0 | 4.0 | 4.0 |
| Monocalcium phosphate | 20 | 20 | 20 |
| Choline chloride | 2.0 | 2.0 | 2.0 |
| Sodium alginate | 20 | 20 | 20 |
| [Zeolite powder](javascript:;) | 10.57 | 6.10 | 1.72 |
| Total | 100.00 | 100.00 | 100.00 |

1. Containing the following (g/kg vitamin premix): thiamine, 0.438; riboflavin, 0.632; pyridoxine^.^HCl, 0.908; *d*-pantothenic acid, 1.724; nicotinic acid, 4.583; biotin, 0.211; folic acid, 0.549; vitamin B-12, 0.001; inositol, 21.053; menadione sodium bisulfite, 0.889; retinyl acetate, 0.677; cholecalciferol, 0.116; *dl*-α-tocopherol-acetate,12.632.
2. Containing the following (g/kg mineral premix): CoCl_2_^.^6H_2_O, 0.074; CuSO_4_^.^ 5H_2_O, 2.5; FeSO_4_^.^7H_2_O, 73.2; NaCl, 40.0; MgSO_4_^.^7H_2_O, 284.0; MnSO_4_^.^H_2_O, 6.50; KI, 0.68; Na_2_SeO_3_, 0.10; ZnSO_4_.7H_2_O, 131.93; Cellulose, 501.09.

**Supplemental Table 2. Ingredients and chemical compositions of diets for adult zebrafish (dry matter, g/kg diet).**

| Ingredient (g/kg diet) | Control diet | Antibiotic diet |
| --- | --- | --- |
| Casein | 400 | 400 |
| Gelatin | 100 | 100 |
| Dextrin | 280 | 280 |
| Soybean oil | 60 | 60 |
| [Lysine](file:///C:\Users\pc\AppData\Local\youdao\dict\Application\7.5.0.0\resultui\dict\?keyword=lysine) | 3.3 | 3.3 |
| VC phosphate | 1.0 | 1.0 |
| Vitamin premix^1^ | 2.0 | 2.0 |
| Mineral premix^2^ | 2.0 | 2.0 |
| Monocalcium phosphate | 20 | 20 |
| Choline chloride | 2.0 | 2.0 |
| Sodium alginate | 20 | 20 |
| Microcrystalline cellulose | 40 | 40 |
| Zeolite powder | 69.7 | 63.9 |
| Polymyxin B | 0.00 | 2.5 |
| Neomycin | 0.00 | 3.3 |
| Total | 1000 | 1000 |
| Proximate analysis | | |
| Crude protein | 42.00 | 42.00 |
| Crude lipid | 6.01 | 6.01 |
| Nitrogen-free extract | 28.00 | 28.00 |
| Gross energy (KJ/g) | 14.02 | 14.02 |

1. Containing the following (g/kg vitamin premix): thiamine, 0.438; riboflavin, 0.632; pyridoxine^.^HCl, 0.908; *d*-pantothenic acid, 1.724; nicotinic acid, 4.583; biotin, 0.211; folic acid, 0.549; vitamin B-12, 0.001; inositol, 21.053; menadione sodium bisulfite, 0.889; retinyl acetate, 0.677; cholecalciferol, 0.116; *dl*-α-tocopherol-acetate,12.632.
2. Containing the following (g/kg mineral premix): CoCl_2_^.^6H_2_O, 0.074; CuSO_4_^.^ 5H_2_O, 2.5; FeSO_4_^.^7H_2_O, 73.2; NaCl, 40.0; MgSO_4_^.^7H_2_O, 284.0; MnSO_4_^.^H_2_O, 6.50; KI, 0.68; Na_2_SeO_3_, 0.10; ZnSO_4_.7H_2_O, 131.93; Cellulose, 501.09.

**Supplemental Table 3. Ingredients and chemical compositions of diets for adult zebrafish (dry matter, g/kg diet).**

| Ingredient (g/kg diet) | Control diet | 0.15% NaAc |  |
| --- | --- | --- | --- |
| Casein | 400 | 400 |  |
| Gelatin | 100 | 100 |  |
| Dextrin | 280 | 280 |  |
| Soybean oil | 60 | 60 |  |
| [Lysine](file:///C:\Users\pc\AppData\Local\youdao\dict\Application\7.5.0.0\resultui\dict\?keyword=lysine) | 3.3 | 3.3 |  |
| VC phosphate | 1.0 | 1.0 |  |
| Vitamin premix^1^ | 2.0 | 2.0 |  |
| Mineral premix^2^ | 2.0 | 2.0 |  |
| Monocalcium phosphate | 20 | 20 |  |
| Choline chloride | 2.0 | 2.0 |  |
| Sodium alginate | 20 | 20 |  |
| Microcrystalline cellulose | 40 | 40 |  |
| Zeolite powder | 69.7 | 68.2 |  |
| Sodium acetate | 0.00 | 1.5 |  |
| Total | 1000 | 1000 |  |
| Proximate analysis | | |  |
| Crude protein | 42.00 | 42.00 |  |
| Crude lipid | 6.01 | 6.01 |  |
| Nitrogen-free extract | 28.00 | 28.00 |  |
| Gross energy (KJ/g) | 14.02 | 14.02 |  |

1. Containing the following (g/kg vitamin premix): thiamine, 0.438; riboflavin, 0.632; pyridoxine^.^HCl, 0.908; *d*-pantothenic acid, 1.724; nicotinic acid, 4.583; biotin, 0.211; folic acid, 0.549; vitamin B-12, 0.001; inositol, 21.053; menadione sodium bisulfite, 0.889; retinyl acetate, 0.677; cholecalciferol, 0.116; *dl*-α-tocopherol-acetate,12.632.
2. Containing the following (g/kg mineral premix): CoCl_2_^.^6H_2_O, 0.074; CuSO_4_^.^ 5H_2_O, 2.5; FeSO_4_^.^7H_2_O, 73.2; NaCl, 40.0; MgSO_4_^.^7H_2_O, 284.0; MnSO_4_^.^H_2_O, 6.50; KI, 0.68; Na_2_SeO_3_, 0.10; ZnSO_4_.7H_2_O, 131.93; Cellulose, 501.09.

**Supplemental Table 4. Ingredients and chemical compositions of diets for adult zebrafish (dry matter, g/kg diet).**

| Ingredient (g/kg diet) | Control diet | 10% Xylose | 1.0% Mannose |
| --- | --- | --- | --- |
| Casein | 400 | 400 | 400 |
| Gelatin | 100 | 100 | 100 |
| Dextrin | 160 | 60 | 150 |
| Xylose/Mannose | 0.00 | 100 | 10 |
| Lard oil | 80 | 80 | 80 |
| Soybean oil | 80 | 80 | 80 |
| [Lysine](file:///C:\Users\pc\AppData\Local\youdao\dict\Application\7.5.0.0\resultui\dict\?keyword=lysine) | 3.3 | 3.3 | 3.3 |
| VC phosphate | 1.0 | 1.0 | 1.0 |
| Vitamin premix^1^ | 4.0 | 4.0 | 4.0 |
| Mineral premix^2^ | 4.0 | 4.0 | 4.0 |
| Calcium dihydrogen phosphate | 20 | 20 | 20 |
| Choline chloride | 2.0 | 2.0 | 2.0 |
| Sodium alginate | 20 | 20 | 20 |
| Microcrystalline cellulose | 40 | 40 | 40 |
| Zeolite powder | 85.7 | 85.7 | 85.7 |
| Total | 1000 | 1000 | 1000 |
| Proximate analysis | | | |
| Crude protein | 42.00 | 42.00 | 42.00 |
| Crude fat | 16.01 | 16.01 | 16.01 |
| Nitrogen-free extract | 16.00 | 16.00 | 16.00 |
| Gross energy (KJ/g) | 15.77 | 15.77 | 15.77 |

1. Containing the following (g/kg vitamin premix): thiamine, 0.438; riboflavin, 0.632; pyridoxine^.^HCl, 0.908; *d*-pantothenic acid, 1.724; nicotinic acid, 4.583; biotin, 0.211; folic acid, 0.549; vitamin B-12, 0.001; inositol, 21.053; menadione sodium bisulfite, 0.889; retinyl acetate, 0.677; cholecalciferol, 0.116; *dl*-α-tocopherol-acetate,12.632.
2. Containing the following (g/kg mineral premix): CoCl_2_^.^6H_2_O, 0.074; CuSO_4_^.^ 5H_2_O, 2.5; FeSO_4_^.^7H_2_O, 73.2; NaCl, 40.0; MgSO_4_^.^7H_2_O, 284.0; MnSO_4_^.^H_2_O, 6.50; KI, 0.68; Na_2_SeO_3_, 0.10; ZnSO_4_.7H_2_O, 131.93; Cellulose, 501.09.

**Supplemental Table 5. Sequences of primers used for *q*PCR analysis.**

| Gene Name | Forward (5’→3’) | Reverse (5’→3’) |
| --- | --- | --- |
| Universal bacteria | CCTACGGGAGGCAGCAG | ATTACCGCGGCTGCTGG |
| Fusobacteria | KGGGCTCAACMCMGTATTGCGT | TCGCGTTAGCTTGGGCGCTG |
| Proteobacteria | TCGTCAGCTCGTGTYGTGA | CGTAAGGGCCATGATG |
| *Cetobacterium* | AGTTTGATCCTGGCTCAGGATG | GAGGCAAGTTCCTTACGCGTT |
| *Plesiomonas* | CTCCGAATACCGTAGAGTGCTATCC | CTCCCCTAGCCCAATAACACCTAAA |
| *Aeromonans* | TCCGGCGGTCTGCACGGCGT | TTGTCCGGGTTGTACTCGTC |
| *rps11* | ACAGAAATGCCCCTTCACTG | GCCTCTTCTCAAAACGGTTG |
| *insulin* | CTGTTGGTCCTGTTGGTCGTGTC | GGGTTGTAGAAGAAGCCTGTTGGG |

**Supplemental Table 6. Ingredients and chemical compositions of diets for larval zebrafish (dry matter, g/kg diet).**

| Ingredient (g/kg diet) | Control diet |
| --- | --- |
| Casein | 460 |
| Gelatin | 110 |
| Dextrin | 180 |
| Lard oil | 30 |
| Soybean oil | 30 |
| Fish liver oil | 20 |
| Soybean lecithin | 20 |
| [Lysine](file:///C:\Users\pc\AppData\Local\youdao\dict\Application\7.5.0.0\resultui\dict\?keyword=lysine) | 1.8 |
| VC phosphate | 1.0 |
| Vitamin premix | 2.0 |
| Mineral premix | 2.0 |
| Monocalcium phosphate | 20 |
| Choline chloride | 2.0 |
| Sodium alginate | 20 |
| [Zeolite powder](javascript:;) | 101.2 |
| Total | 1000 |
| Proximate analysis | |
| Crude protein | 48.00 |
| Crude lipid | 9.90 |
| Gross energy (KJ/g) | 18.60 |

1. Containing the following (g/kg vitamin premix): thiamine, 0.438; riboflavin, 0.632; pyridoxine^.^HCl, 0.908; *d*-pantothenic acid, 1.724; nicotinic acid, 4.583; biotin, 0.211; folic acid, 0.549; vitamin B-12, 0.001; inositol, 21.053; menadione sodium bisulfite, 0.889; retinyl acetate, 0.677; cholecalciferol, 0.116; *dl*-α-tocopherol-acetate,12.632.
2. Containing the following (g/kg mineral premix): CoCl_2_^.^6H_2_O, 0.074; CuSO_4_^.^ 5H_2_O, 2.5; FeSO_4_^.^7H_2_O, 73.2; NaCl, 40.0; MgSO_4_^.^7H_2_O, 284.0; MnSO_4_^.^H_2_O, 6.50; KI, 0.68; Na_2_SeO_3_, 0.10; ZnSO_4_.7H_2_O, 131.93; Cellulose, 501.09.

**Supplemental Table 7.** **Diversity index of zebrafish gut microbiota fed with CD, OD and HD for two weeks.^1^**

| Sample | ASVs | ACE | Chao1 | Simpson | Shannon |
| --- | --- | --- | --- | --- | --- |
| CD | 60.25±5.20 | 60.25±5.20 | 60.25±5.20 | 0.88±0.03 | 4.28±0.26 |
| OD | 79.50±19.36 | 79.50±19.36 | 79.50±19.36 | 0.91±0.005 | 4.58±0.23 |
| HD | 127.00±28.69 | 127.00±28.69 | 127.00±28.69 | 0.91±0.019 | 5.12±0.42 |

^1^Values are expressed as the mean ± SEM, n = 4. ASVs, amplicon sequence variants; Chao1, Chao1 index; ACE, ACE index; Simpson, Simpson’s diversity index; Shannon, Shannon diversity index.

**Supplemental Table 8. The predominant gut bacterial species in zebrafish fed CD, OD and HD for two weeks.^1^**

| Species (%) | CD | OD | HD |
| --- | --- | --- | --- |
| *Cetobacterium somerae* | 1.0±0.6^b^ | 8.0±3.0^a^ | 3.0±2.0^ab^ |
| *Plesiomonas shigelloides* | 47±13^a^ | 14±5.0^a^ | 0.06±0.03^b^ |
| *Enterobacter sp.* | 2.0±0.9 | 6.0±.3.0 | 15±6.0 |
| *Enterobacter cloacae* | 3.0±2.0 | 13±5.0 | 5.0±2.0 |
| *Rhizobium sp.* | 7.0±3.0^b^ | 19±7.0^ab^ | 27±2.8^a^ |
| *Fimbriiglobus sp.* | 0.3±0.2^b^ | 4.0±2.0^ab^ | 6.0±0.5^a^ |
| *Aquitalea magnusonii* | 9.5±3.6^a^ | 0.4±0.2^b^ | 0.07±0.06^c^ |
| *Bacillus circulans* | 2.0±1.0 | 3.0±.2.0 | 6.0±1.0 |
| *Bosea sp.* | 3.0±5.0 | 0.9±0.4 | 2.0±0.4 |
| *Singulisphaera sp.* | 0.6±0.6^b^ | 1.4±0.5^b^ | 4.0±1.0^a^ |
| Others | 24±4.0 | 21±3.0 | 41±7.0 |

^1^Values are expressed as the mean ± SEM, n = 4. Means marked with different letters represent statistically significant results (*P* < 0.05), whereas the same letter corresponds to results that show no statistically significant differences.

**Supplemental Table 9. The results of biochemical reaction analysis of *C. somerae.***

| Substrate | *C. somerae* | | *P. shigelloides* | | [*A.*](javascript:;)*hydrophila* NJ-1 | | [*A.*](javascript:;)*hydrophila* CI098 | |
| --- | --- | --- | --- | --- | --- | --- | --- | --- |
|  | Anaerobic | Anaerobic | | Aerobic | Anaerobic | Aerobic | Anaerobic | Aerobic |
| Saccharose | +/+ | -/- | | -/- | +/+ | +/+ | +/+ | +/+ |
| β-galactoside | -/- | +/+ | | +/+ | (+)/(+) | +/+ | (+)/(+) | +/+ |
| Mannose | +/+ | -/- | | -/- | +/+ | +/+ | +/+ | +/+ |
| Galactose | (+)/(+) | +/+ | | +/+ | +/+ | +/+ | +/+ | +/+ |
| Rhamnose | -/- | -/- | | -/- | -/- | -/- | -/- | -/- |
| Glucose | +/+ | +/+ | | +/+ | +/+ | +/+ | +/+ | +/+ |
| Arabinose | (+)/(+) | (+)/- | | (+)/- | +/+ | +/+ | +/+ | +/+ |
| Cellose | -/- | (+)/- | | (+)/(+) | -/- | -/- | +/+ | (+)/(+) |
| Esculin | -/- | (+)/(+) | | -/- | +/+ | +/+ | -/- | -/- |
| Fructose | +/+ | +/+ | | (+)/(+) | +/+ | +/+ | +/+ | +/+ |
| Amygdalin | (+)/(+) | -/- | | (+)/(+) | -/- | -/- | -/- | -/- |
| Glucosamine | +/+ | +/+ | | +/+ | +/+ | +/+ | +/+ | +/+ |
| Gluconate | -/- | -/- | | -/- | -/- | -/- | -/- | -/- |
| Lactose | -/- | (+)/- | | (+)/(+) | -/- | -/- | -/- | -/- |
| Maltose | +/+ | +/+ | | +/+ | +/+ | +/+ | +/+ | +/+ |
| Dextrin | (+)/(+) | (+)/(+) | | (+)/(+) | +/+ | +/+ | +/+ | +/+ |
| Mannitol | -/- | -/- | | -/(+) | +/+ | +/+ | +/+ | (+)/(+) |
| Ribose |  | -/- | | -/- | -/- | -/- | -/- | -/- |
| Starch | +/+ | +/+ | | +/+ | -/- | + |  | + |
| Melezitose | -/- | -/- | | -/- | -/- | -/- | -/- | -/- |
| Melibiose | (+)/+ | -/- | | (+)/(+) | -/- | -/- | +/+ | +/+ |
| Raffinose | (+)/(+) | -/- | | (+)/(+) | -/- | -/- | +/+ | +/+ |
| Mycose | +/+ | +/+ | | +/+ | +/+ | (+)/(+) | +/+ | +/+ |
| Saligenin | -/- | - | | (+) | +/+ | +/+ | -/- | -/- |
| Sorbitol | -/- | -/- | | (+)/(+) | -/- | -/- | -/- | -/- |
| Xylose | +/+ | -/- | | (+)/(+) | -/- | -/- | -/- | -/- |

The results were shown positive with “+”, weak positive with “(+)”, negative with “-”.
